# Supplementary material for: Evaluations of psychosocial cancer support services: A scoping review
Source: PLoS One. 2021 May 4;16(5):e0251126. doi: 10.1371/journal.pone.0251126 (PMC8096026; doi:10.1371/journal.pone.0251126)
Supplement: S1 Table — (DOCX) [file pone.0251126.s001.docx]

S1 Table.

Pubmed complete search example

|  | **CODE** |
| --- | --- |
| 1 | ((((cancer[Title]) AND counselling[Title]) AND center*[Title/Abstract]) NOT genetic*[Title]) NOT therap*[Title] |
| 2 | ((((cancer[Title]) AND counselling[Title]) AND centre*[Title/Abstract]) NOT genetic*[Title]) NOT therap*[Title] |
| 3 | ((((cancer[Title]) AND counseling[Title]) AND center*[Title/Abstract]) NOT genetic*[Title]) NOT therap*[Title] |
| 4 | ((((cancer[Title]) AND counseling[Title]) AND centre*[Title/Abstract]) NOT genetic*[Title]) NOT therap*[Title] |
| 5 | cancer[Title] AND service[Title] AND center*[Title] |
| 6 | cancer[Title] AND service[Title] AND centre*[Title] |
| 7 | cancer[Title] AND support[Title] AND center*[Title] |
| 8 | cancer[Title] AND support[Title] AND centre*[Title] |
| 9 | cancer[Title] AND support[Title] AND service*[Title] |
| 10 | Cancer counselling[Title/Abstract] |
| 11 | Cancer counseling[Title/Abstract] |
| 12 | Psychosocial care[Title] AND cancer[Title] |
| 13 | Psychosocial counseling[Title/Abstract] AND cancer[Title/Abstract] |
| 14 | Psychosocial counselling[Title/Abstract] AND cancer[Title/Abstract] |
| 15 | Outpatient cancer care[Title/Abstract] |
| 16 | Supportive psychotherapy[Title/Abstract] |
| 17 | 1 OR 2 OR 3 OR 4 OR 5 OR 6 OR 7 OR 8 OR 9 OR 10 OR 11 OR 12 OR 13 OR 14 OR 15 OR 16 |
