# Supplementary material for: Evaluations of psychosocial cancer support services: A scoping review
Source: PLoS One. 2021 May 4;16(5):e0251126. doi: 10.1371/journal.pone.0251126 (PMC8096026; doi:10.1371/journal.pone.0251126)
Supplement: Attachment — Submitted filename: Response to Reviewers.docx [file pone.0251126.s003.docx]

**Response to Reviewers**

Reviewer #1: Thank you again for you previous comments and for accepting the manuscript!

Reviewer #2: The authors have done a substantial amount of work to address previous reviewer comments, much of which has helped improve the manuscript. However, some of the additions appear to be rushed and not well thought-through. The lack of editing and refinement in some of these sections (particularly in the introduction) make it hard to follow. Based on the updated manuscript, I have outlined some further modifications that I think may help improve this work.

Abstract
- Rather than ‘most studies’ and ‘Few studies’, please list the number for each.

Reply: Thank you. We have made the changes as you suggested.

- Please change ‘the support services evaluated’ to ‘the evaluated support services’.

Reply: We agree and have adjusted the wording.

- Given the low number of studies and lack of RCTs, I think the results statement needs to be more tentative. For instance, ‘while the included studies indicate some improvements to well-being for patients with cancer, the low number and lack of high quality of studies indicate these findings should be interpreted with caution.’

Reply: That is a good suggestion, so we have included your formulation in the abstract.

- Suggest removing the last statement. Don’t only need more high quality studies for this, but also need high quality studies to determine that the interventions are effective. This statement is a jump in logic.

Reply: We have deleted the last statement and strengthened the conclusion as proposed by you.

Introduction
- First sentence of paragraph 2 could be refined, for example – ‘A cancer diagnosis can have a substantial impact on an individual’s mental health and wellbeing, which may require diverse psychological and social support’.

Reply: We believe your formulation is more precise and have edited the first sentence accordingly.

- Replace ‘form of delivery’ with ‘delivery format’

Reply: Agree!

- Not sure I understand the relevance of the sentence on Pg 5, lines 75-76 (professionals from different fields etc.).

Reply: We have deleted the sentence to improve the clarity of the paragraph.

- The additional sections of the introduction, specifically the second and third paragraphs are difficult to follow. The paragraphs lack refinement and the logic is not clear. The second paragraph focuses on the flexibility of services though the relevance of this information is not established. There appears to be a vague link in the third paragraph where there is mention of outpatient settings having differing content etc. but again the link to the current paper isn’t clear. I think these sections would benefit from greater thought and refinement.

Reply: Thank you for your remark. We agree that the point we are trying to make, could be clearer. Thus, we have combined paragraph 2 and 3 and strengthened the line of argumentation here:

“There is some indication of the efficacy of psychosocial support for patients with cancer in improving the quality of life, decreasing levels of distress and the risk for developing depression or anxiety disorders (11-15). The interventions tested for efficacy and summarized in the reviews are often manualized or tested in standardized settings for the purpose of the studies. However, the interventions often need to be adapted according to the diverse needs of patients with cancer when implemented in the health care setting (16-18). Hence, implemented psychosocial interventions may be flexible in duration, contents, delivery format (e.g., inpatient or outpatient setting and online, face-to-face or telephone) and may be administered by different health care staff (e.g., psychologists, nurses, trained volunteers) (8, 16). The efficacy of psychosocial interventions investigated in a scientific study may not be transferable to implemented psychosocial support since some aspects may change with different types, contexts and set-ups of the support services. Therefore evaluations of implemented psychosocial support in out-patient health care are important to assess the effectiveness considering their ecological validity and quality standards. The current literature and reviews on the topic reveal a scarcity of evaluations of implemented psychosocial support.“ (line 58-72)

- In the last paragraph of the introduction (page 6) – could the authors remove ‘we’ from all sentences to make the sentences objective. E.g. ‘For this review psychosocial cancer support services are defined as…’

Reply: We have deleted ‘we’ from all sentences.

- Similar to above, the last sentence requires refinement – ‘A scoping review will provide an overview of the available literature to guide future evaluations of this evidence base’…or similar. Indicating why scoping reviews are useful and specifically why one is needed on this topic would be preferential to the current justifications.

Reply: We have made the last sentence more precise. We have not added any further justifications for choosing a scoping review, as the mere definition of scoping reviews is to provide a first overview of the available literature, which we have stated.

Methods
- Please provide the references to the reviews that have already been conducted on page 7, lines 126-127.

Reply: We have included the references.

- The justification that previous studies have examined telephone and online interventions is sufficient for exclusion and more compelling than the justification re the possible inclusion of inpatients.

Reply: We agree and have edited the section accordingly.

- Page 7, lines 128-130: This sentence is difficult to follow. Indicating that supportive psychotherapy was excluded from your exclusion criteria for psychotherapy is a confusing way to present this information.

Reply: We understand how it could be confusing. Therefore, we deleted ‘supportive psychotherapy’ from the section and mentioned it in the inclusion criteria section.

- Page 8, line 151 – remove ‘journal’ from the ‘information on the article’ list.

Reply: Thank you for noticing. We have removed ‘journal’.

Results
- Page 19, line 233 – think there is a typo here. Perhaps ‘measured’ should be ‘measures’?

Reply: Yes, you are right. It is supposed to be ‘measures’.

- Page 26, lines 240-244 – long, convoluted sentence. Difficult to follow.

Reply: We have shortened the sentence to eliminate any confusion: *“The results of the RCT showed that physical well-being improved after the support compared to the control group but not psychological and social well-being”*

- Page 26, line 244-245 – Needs revising, perhaps ‘All X pre and post studies using the MYCaW demonstrated improvements in general well-being’.

Reply: Thank you. The sentence now reads as follows: *“All four pre- and post- studies using the MYCaW demonstrated improvement in general well-being”*

- Rather than ‘one study’ could state the authors name followed by et al.

Reply: Thank you, we have included the name.

- Page 26, line 247 – consider replacing ‘revealed’ with ‘demonstrated’ or ‘found’.

Reply: We have replaced it with ‘found’.

- Page 26, line 252 – ‘All articles’ appears to refer to a subset of articles (perhaps those that examined concerns?). Please clarify.

Reply: Yes, we’ve meant all articles that examined concerns and have added this information.

Discussion
- Page 27, lines 269-271 – I’m not sure I understand why the heterogeneity of the psychosocial support would result in a lack of RCTs? Also when you say it is difficult to have a control group when implementing an intervention in health care, is this attempting to suggest that withholding support would be unethical in practice? Please clarify.

Reply: You are right. The section lacks clarity. We have strengthened the section and explanation: *“The lack of studies with RCT designs may be due to the lack of standardized interventions offered in psychosocial support, which may hamper comparability. Another reason may be the difficulty of integrating a control group when assessing an intervention already implemented in health care, since it would be unethical to deny support for patients in need. One solution could be waitlist-control trials.”* (line 213-217)

- Page 27, line 276-279 – The review does not examine actual support services, it examined evaluation of services (i.e. research studies). Stating that support services are more prevalent in certain countries and approached by more women than men is not a finding of this research. It should be stated that the evaluations were conducted primarily in Europe and North America and that a higher number of women than men participated in these studies etc.

Reply: You are correct, we examine evaluations of support services. However, we believe that it is valuable to also discuss the findings of those evaluations. Nevertheless we agree, that we need to clarify that these are not direct findings and that there are several ways to interpret the results. The origin of the studies may be due to a stronger focus of research in the field for those countries, which again may be i.e., due to resources available. On the contrary, it may also be the case that psychosocial support services are more common in those countries and thus there may be more need for research. We have edited this section in the discussion so it becomes clearer, that there are different interpretation to the findings.

- Similar to above – this review didn’t examine whether women were more likely to seek help but rather observed a higher number of women participating in psychosocial support evaluations. This may reflect a greater willingness of women to seek support – but this should be tentative and not presented as a direct finding since it may also be due to study recruitment strategies (e.g. targeting breast cancer) or women being more likely to participate in research studies.

Reply: Similar as above – we agree, it should not be presented as direct finding. It may be due to the study design or approach that across all studies more women were included because women are more likely to participate in studies. Since we assume that most studies considered the external validity and generalizable of their sample, we believe that it can be valuable to discuss the results and trends of the studies evaluating support services.

- Page 28, lines 289-292 – this sentence needs to be edited for clarity.

Reply: We have changed the sentence to improve the clarity: “*Two support services targeted specifically breast cancer patients and ten support services offered support for patients with any cancer diagnosis, which is also in line with the findings of a study assessing the characteristics of patients in multiple cancer support centers*” (line 245-247).

- Page 29, lines 321-325 – These statements are a bit unclear. Are the authors saying the measure examine concerns followed by well-being, which may bias the participant responses to rate their well-being higher?

Reply: Yes, this is what we indented to express with the sentences. We clarified the section: *“General and psychological well-being measured by the MYCaW and FACIT-SpEx resulted in clinically significant improvement (26, 29-31). The improvement of well-being when measured by the MYCaW may be due to the proximity of its assessment with that of the concerns (Concerns are examined followed by well-being). The patient may connect the successful resolution of the concerns with an improvement of well-being. Further research is needed to study the improvement of well-being when assessed in combination with concerns in the setting of psychosocial support.”* (line 265-272)

- Page 30, lines 359-361 – as with the introduction, please remove reference to ‘we decided’. Rather provide the scientific rationale behind providing methodological coding. E.g. ‘While a scoping review does not require quality assessment, this was included for the current review to provide greater insight on the limited number of studies.’

Reply: Yes, thank you! We like your suggestion and have exchanged it.

- Information on the EPHPP (i.e. what it assessed and how the rating works) should be included in methods not discussion.

Reply: We agree with you that the methodological part of the discussion needs to be moved to the method and the discussion of the EPHPP should be at the beginning of the discussion. (See line 128-135 and line 222-228)

- I would also suggest talking about the methodological findings prior to the study findings in the discussion, as this sets up the reader to understand the quality of the studies being presented.

Reply: See our comment above.

- Page 31, line 386 – interpreting the origin of the research does not need to be done with caution, the origin of the research is clearly stated in the research articles. Suggest removing this statement.

Reply: Yes we agree, that sentence is irritating so we have deleted it.

- Replace ‘gives rise to’ with ‘highlights’.

Reply: We thank you for the suggestion and have changed it.

- Conclusion – as per comment about the abstract, there is a jump in logic for improving evidence would increase funding. Rather clear evidence indicating that psychosocial support services improve outcomes for patients with cancer may result in increased funding.

Reply: We agree that there is a jump in logic and adjusted the conclusion accordingly.
