# Supplementary material for: Evaluations of psychosocial cancer support services: A scoping review
Source: PLoS One. 2021 May 4;16(5):e0251126. doi: 10.1371/journal.pone.0251126 (PMC8096026; doi:10.1371/journal.pone.0251126)
Supplement: Attachment — Submitted filename: Response to Reviewers.docx [file pone.0251126.s004.docx]

**Response to Reviewers**

**Reviewer #2**: Thank you for the updates to the manuscript. I suggest the minor editorial updates listed below and then accept the manuscript. Well done.

**Reply:** Thank you very much! We have incorporated the changes as you suggested below.

Pg 4. Ln 63-64. Please modify to: The interventions tested for efficacy and summarized in the reviews are largely controlled and standardized to reduce the likelihood of experimental bias.
Pg 4. Ln 69-70. Please modify to: The efficacy of psychosocial interventions investigated in an experimental study may not be transferable when implemented to real-world psychosocial support services, due to the different settings and contexts of the support services.
Pg 4. Ln 72-73. Please modify to: …assess their effectiveness with consideration to their ecological validity and quality.
Pg 4. Ln 73-74. Please modify to: There is currently no evaluation of implemented psychosocial support within out-patient cancer settings.
Pg 5. Ln 97-98. Please modify to: This review focuses on face to-face delivered support since overviews of telephone and online support has been reported elsewhere (19-21).
Pg 5. Ln 117-118. Please modify to: Studies evaluating psychotherapy aimed at treating mental disorders were excluded.
Pg 7. Ln 155: Please delete “It is important to note that”.
Pg 17. Ln 212: Please change “support” to “supportive intervention”. Further, please change latter part of sentence to: “but psychological and social well-being did not”.
Pg 18. Ln 242-243” Please move sentence to the end of the paragraph so it reads: “One possible study design to overcome this concern could involve using wait-list controls”.
Pg 18. Ln 249: Replace “mainly” with “largely”
Pg 19. Ln 265-267: Please modify to: Greater participation of women than men in the evaluated studies may be due to increased willingness of women to participate in research studies.

**Responses to Editor**

Additional Editor Comments (if provided):

Please insert headings into the Abstract including Background, Methods, Results and Conclusions.

**Reply:** We included the headings as suggested.

In the manuscript, please make changes as follows:

Methods

- Insert the heading Quality Assessment or Risk of Bias before discussing the EPHPP;

**Reply:** We have inserted the heading quality assessment.

- Further develop the synthesis section to explain why a meta-analysis was not considered possible where the same measure was used in multiple studies (presumably related to the statement in the Discussion that "the heterogeneity of the services and their offers makes it difficult to interpret and compare the outcomes across the selected studies").

- Please also include a brief description of the narrative approach taken to comparing results across studies.

**Reply:** Thank you for the request. We have included an explanation as suggested for the choice against a meta-analysis and our narrative approach to comparing the results:

“For articles that used the same instruments a meta-analysis would be the preferable choice. However, due to the heterogeneity of the setting and context of psychosocial support services, comparability is not guaranteed. Hence, a scoping review was the reasonable alternative.” (line 136-138)

“Furthermore, since different instruments were used for the evaluation of services, the results were compared across studies within similar theoretical concepts e.g. satisfaction with the service. The global scores and details of the quality assessment are reported in Table 1.”
(line 142-145)

Results

- I think Table 3 can be deleted and the final column moved to Table 2. Table 2 already includes details of measures, and the validity/reliability columns are themselves neither valid nor reliable given that these are continuous rather than dichotomous variables. To make more space in Table 2, I recommend moving the quality rating to a new table where rating of each characteristic is reported as well as the overall grade.

**Reply:** Thank you, we have deleted table 3 and added the last column to table 2. We included the quality rating (previously in the supporting information S3 Table) as a new table (Table 1) in the manuscript.

Discussion

Please remove the sub-heading 'summary of evidence'.

**Reply:** We have removed the subheading.

I think the author's have made assumptions regarding the nature of scoping vs systematic reviews that are not justified given the various ways in which the former term is used. Please remove the following sentences:

"As the small number of suitable articles suggests, the scoping review format was a reasonable choice"

"While a scoping review does not require a quality assessment, this was included for the 222 current review to provide greater insight on the limited number of studies" (changing the sentence that follows to clarify it is referring to quality rating).

**Reply:** We have deleted the sentences as suggested.

**Journal Requirements:**

Please review your reference list to ensure that it is complete and correct. If you have cited papers that have been retracted, please include the rationale for doing so in the manuscript text, or remove these references and replace them with relevant current references. Any changes to the reference list should be mentioned in the rebuttal letter that accompanies your revised manuscript. If you need to cite a retracted article, indicate the article’s retracted status in the References list and also include a citation and full reference for the retraction notice.

**Reply:** The retracted article has been removed:

Gordon VM, Madhotra R, Steer P. A Single Centre Experience of Straight to Test Suspected Ugi Cancer Service. Gut. 2011;60.

Changes that have been made through-out the revision process to the reference list are listed below:

**Removed:**

Cousson-Gelie F, Bruchon-Schweitzer M, Atzeni T, Houede N. Evaluation of a Psychosocial Intervention on Social Support, Perceived Control, Coping Strategies, Emotional Distress, and Quality of Life of Breast Cancer Patients. Psychol Rep. 2011;108(3):923-42.

Boltong A, Ledwick M, Babb K, Sutton C, Ugalde A. Exploring the rationale, experience and impact of using Cancer Information and Support (CIS) services: an international qualitative study. Support Care Cancer. 2017;25(4):1221-8.

Öhlén J, Holm A-K, Karlsson B, Ahlberg K. Evaluation of a counselling service in psychosocial cancer care. Eur J Oncol Nurs. 2005;9(1):64-73.

Jevne RF. Looking back to look ahead: A retrospective study of referrals to a cancer counselling service. International Journal for the Advancement of Counselling. 1990;13(1):61-72.

Furzer BJ, Wright KE, Petterson AS, Wallman KE, Ackland TR, Joske DJ. Characteristics and quality of life of patients presenting to cancer support centres: patient rated outcomes and use of complementary therapies. BMC Altern Med. 2013;13:169.

**Included:**

Holland JC, Andersen B, Breitbart WS, Compas B, Dudley MM, Fleishman S, et al. Distress Management Clinical Practice Guidelines in Oncology (TM). J Natl Compr Canc Ne. 2010;8(4):448-85.

Morris SB. Estimating effect sizes from pretest-posttest-control group designs. Organ Res Methods. 2008;11(2):364-86.

Lebow J. Overview of psychotherapies UpToDate Inc.: Waltham, MA, USA.2019 [Available from: <http://www.uptodate.com>.

Page AE, Adler NE. Cancer care for the whole patient: Meeting psychosocial health needs: National Academies Press; 2008.

Watson M, Kissane DW. Handbook of Psychotherapy in Cancer Care. 1 ed: John Wiley & Sons, Inc.; 2011.

Gabriel I, Creedy D, Coyne E. A systematic review of psychosocial interventions to improve quality of life of people with cancer and their family caregivers. Nurs Open. 2020;7(5):1299-312.

Galway K, Black A, Cantwell M, Cardwell CR, Mills M, Donnelly M. Psychosocial interventions to improve quality of life and emotional wellbeing for recently diagnosed cancer patients. Cochrane Db Syst Rev. 2012(11).

Salsman JM, Pustejovsky JE, Schueller SM, Hernandez R, Berendsen M, McLouth LES, et al. Psychosocial interventions for cancer survivors: A meta-analysis of effects on positive affect. J Cancer Surviv. 2019;13(6):943-55.

Teo I, Krishnan A, Lee GL. Psychosocial interventions for advanced cancer patients: A systematic review. Psycho-Oncology. 2019;28(7):1394-407.

Vartolomei L, Shariat SF, Vartolomei MD. Psychotherapeutic Interventions Targeting Prostate Cancer Patients: A Systematic Review of the Literature. European urology oncology. 2018;1(4):283-91.

Lederberg MS, Holland JC. Supportive psychotherapy in cancer care: An essential ingredient of all therapy. Handbook of psychotherapy in cancer care. 2011;1.

Boltong A, Ledwick M, Babb K, Sutton C, Ugalde A. Exploring the rationale, experience and impact of using Cancer Information and Support (CIS) services: an international qualitative study. Support Care Cancer. 2017;25(4):1221-8.

Hong Y, Pena-Purcell NC, Ory MG. Outcomes of online support and resources for cancer survivors: A systematic literature review. Patient Educ Couns. 2012;86(3):288-96.

Okuyama S, Jones W, Ricklefs C, Tran ZV. Psychosocial telephone interventions for patients with cancer and survivors: a systematic review. Psycho-Oncology. 2015;24(8):857-70.

Munn Z, Peters MDJ, Stern C, Tufanaru C, McArthur A, Aromataris E. Systematic review or scoping review? Guidance for authors when choosing between a systematic or scoping review approach. Bmc Med Res Methodol. 2018;18.

Baker BS, Hoffman CJ, Fenlon D. What can a third sector organisation provide for people with breast cancer that public health services cannot? Developing support services in response to service evaluation. Eur J Integr Med. 2019;30:9.

Grassi L, Fujisawa D, Odyio P, Asuzu C, Ashley L, Bultz B, et al. Disparities in psychosocial cancer care: a report from the International Federation of Psycho-oncology Societies. Psycho-Oncology. 2016;25(10):1127-36.

Mackenzie CS, Gekoski WL, Knox VJ. Age, gender, and the underutilization of mental health services: The influence of help-seeking attitudes. Aging & Mental Health. 2006;10(6):574-82.

Merckaert I, Libert Y, Messin S, Milani M, Slachmuylder JL, Razavi D. Cancer patients' desire for psychological support: prevalence and implications for screening patients' psychological needs. Psychooncology. 2010;19(2):141-9.

Gessler S, Low J, Daniells E, Williams R, Brough V, Tookman A, et al. Screening for distress in cancer patients: is the distress thermometer a valid measure in the UK and does it measure change over time? A prospective validation study. Psycho-Oncology. 2008;17(6):538-47.

Doherty M, Miller-Sonet E, Gardner D, Epstein I. Exploring the role of psychosocial care in value-based oncology: Results from a survey of 3000 cancer patients and survivors. J Psychosoc Oncol. 2019;37(4):441-55
