# Supplementary material for: Evaluations of psychosocial cancer support services: A scoping review
Source: PLoS One. 2021 May 4;16(5):e0251126. doi: 10.1371/journal.pone.0251126 (PMC8096026; doi:10.1371/journal.pone.0251126)
Supplement: Attachment — Submitted filename: Response to Reviewers.docx [file pone.0251126.s005.docx]

**Response to Reviewers**

Academic editor:
Specifically, please replace "scoping review" with "narrative synthesis" in the following sentence: "Hence, a scoping review was the reasonable alternative

**Reply:** We edited the sentence as suggested.
